# Conditional cell reprogramming for modeling host‐virus interactions and human viral diseases

**DOI:** 10.1002/jmv.26093

**Published:** 2020-06-16

**Authors:** Xuefeng Liu, Abdul M. Mondal

**Affiliations:** ^1^ Department of Pathology, Center for Cell Reprogramming Georgetown University Medical Center Washington DC; ^2^ Department of Oncology, Lombardi Comprehensive Cancer Center Georgetown University Medical Center Washington DC

**Keywords:** air‐liquid interface, cell senescence, conditional reprogramming, emerging viruses, functional models, normal cells, organoids, physiological conditions

## Abstract

Conventional cancer and transformed cell lines are widely used in cancer biology and other fields within biology. These cells usually have abnormalities from the original tumor itself, but may also develop abnormalities due to genetic manipulation, or genetic and epigenetic changes during long‐term passages. Primary cultures may maintain lineage functions as the original tissue types, yet they have a very limited life span or population doubling time because of the nature of cellular senescence. Primary cultures usually have very low yields, and the high variability from any original tissue specimens, largely limiting their applications in research. Animal models are often used for studies of virus infections, disease modeling, development of antiviral drugs, and vaccines. Human viruses often need a series of passages in vivo to adapt to the host environment because of variable receptors on the cell surface and may have intracellular restrictions from the cell types or host species. Here, we describe a long‐term cell culture system, conditionally reprogrammed cells (CRCs), and its applications in modeling human viral diseases and drug discovery. Using feeder layer coculture in presence of Y‐27632 (conditional reprogramming, CR), CRCs can be obtained and rapidly propagated from surgical specimens, core or needle biopsies, and other minimally invasive or noninvasive specimens, for example, nasal cavity brushing. CRCs preserve their lineage functions and provide biologically relevant and physiological conditions, which are suitable for studies of viral entry and replication, innate immune responses of host cells, and discovery of antiviral drugs. In this review, we summarize the applications of CR technology in modeling host‐virus interactions and human viral diseases including severe acute respiratory syndrome coronavirus‐2 and coronavirus disease‐2019, and antiviral discovery.

## 1 CELL MODELS

Conventional cell lines have been widely used for many aspects of biomedical research. The most famous case is HeLa cells, the first human cell line, derived from the cervical cancer tissue of Henrietta Lacks in 1951. Around 70 000 scientific articles encompassing almost every conceivable aspect of the study have been published, for example, to understand the basic machinery of normal and diseased cell biology. Although cancer cell lines have been useful for in vitro experiments to study cancer biology and discovery of novel biomarkers and targets, drawing conclusions to clinical oncology is still challenging as cancer cell lines are usually clonal during in vitro passage and lack the cellular heterogeneity and complexity of human cancers. The current available cancer cell lines cannot reflect the spectrum of cancer cell types, or cancers from diverse racial and ethnic groups, or genetic background of the patients where they are derived. Several exciting technologies for patient‐derived cancer models (PDCMs) have been developed for next generation of cancer models, including organoids, induced pluripotent stem cells (iPSCs), patient‐derived xenografts (PDXs), and conditionally reprogrammed cells (CRCs).^1^ However, cancer cells have a distinct physiology that is very different from normal cells in the tissue, and differs in how cells send or receive signals to or from their neighboring cells. There are usually several different cell types in a normal tissue because of cellular diversity and introduce the question: how do all DNAs, RNAs, proteins, other molecules in a cell act together to define the properties of other cell types? Furthermore, cancer cells cannot distinguish one cell type from another, for example, a lung cell from a kidney cell. How do the normal cells in various tissues respond to external stimuli and exposures? For example, response to a virus infection and exposure to different environmental factors. To answer this question, we need to make the difficult transition from the study of cancer cells and all the research tools that have been developed in cell biology and disease modeling to normal cells. Obviously, primary cells are most appropriate for these purposes. However, primary cells have a very limited life span or population doubling time due to the nature of cell senescence, and they also have very low yields and high variability from any original tissue specimens. As PDCM described above, organoids, iPSCs, and CRCs have been used for the generation or expansion of human normal cells, and these serve as great resources for cell biology and modeling human diseases including viral infections and antiviral discovery.^1^, ^2^ We summarize the characteristics of these approaches in Table 1. In this review, we focus on the applications of CRCs in modeling host‐virus interactions and human viral diseases, and antiviral discovery.

**Table 1:** In vitro model systems for viral diseases

	Cancer cells	Transformed/immortalized cells	iPS cells	Organoids	CR cells	Primary cells
Sample size	(++++)	(+++)	(++)	(++)	(+)	(+++)
Timing	1‐5 mo	1‐2 mo	2‐10 wk	1‐4 wk	1‐10 d	1‐4 wk
Success rate	Extremely low	Medium	Medium	High	High	Low
Rapid expansion	High	High	Medium	Medium	High	Low
Genetic stability	Low	Low	Medium	High	High	High
Cost	Low	Low	Medium	High	Low	High
HT screening	(++++)	(++++)	(+)	(++)	(++++)	(+)
Physiology	Low	Low	Medium	High	High	High
Life span	Long	Long	Long	Long	Long	Very limited
Difficulty of differentiation	(++++)	(++++)	(+++)	(+)	(+)	(+)
Biobanking	(−)	(+)	(++++)	(++++)	(++++)	(+)
Tissue specific	Low	Low	Low	High	High	High
Genetic manipulation	Yes	Yes	Yes	Yes	Yes	No

## 2 CONDITIONAL CELL REPROGRAMMING

### 2.1 CR is rapid

We initially discovered that combination of feeder layers and a Rho kinase inhibitor, Y‐27632, allows the generation of long‐term cultures of both normal and tumor cells from keratinocyte and non‐keratinocytes tissues.^3^, ^4^, ^5^, ^6^ The culture condition may convert or reprogram the whole‐cell populations in synthetic medium to a stem cell‐like status within 2 days, rather than a long‐term clonal selection.^3^, ^4^ These Reprogrammed cells quickly stop proliferating or differentiate after removal of one of the culture conditions, either Y‐27632 or feeder layer. Usually typical epithelial cell colonies are surrounded by feeder cells that can be visualized within 18 to 36 hours after initial plating from single‐cell suspension. Thus, we termed this cell technology as “conditional reprogramming (CR),” and the resulting cells as “conditionally reprogrammed cells (CRC),” respectively.^1^, ^3^, ^4^, ^5^, ^6^ As normal CRCs maintain their lineages and differentiation functions under in vitro three‐dimensional (3D) or *in vivo* conditions, the CR technology has been widely used in basic and translational cancer biology, disease modeling, tissue regeneration, evaluation of drug toxicity, virus infections, and so on. Indeed, organoids^7^, ^8^, ^9^, ^10^, ^11^ and CR technologies have been both recognized as the key new technologies by NIH precision oncology,^12^, ^13^ and have also been used for human cancer model initiatives (HCMI) program with ATCC (https://www.atcc.org/en/Products/Cells_and_Microorganisms/HCMI.aspx?utm_id=t18020438l1).

### 2.2 CR technology is robust

**Figure 1:**
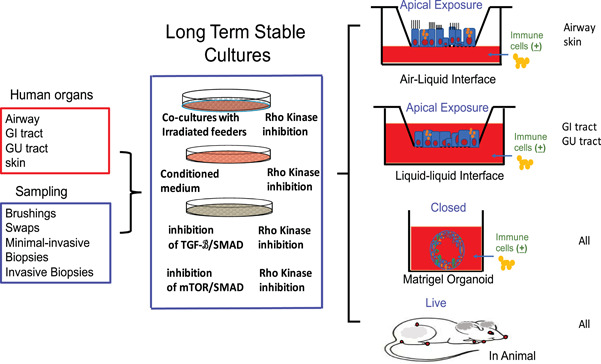
Workflow of normal CRC cultures from non‐ or minimally invasive biopsies and physiological differentiation models under in vitro apical (ALI and LLI) or closed (organoids) 3D cultures, and in vivo (in animal). ALI, air‐liquid interface; CRC, conditionally reprogrammed cells; LLI, liquid‐liquid interface

Most model technologies need large materials to begin with, for example, the establishment of PDX models for human tumor studies usually require surgical specimens. CR technology allows the generation of cell cultures from surgical specimens, core or needle biopsies, and other minimally invasive or noninvasive specimens, for example, nasal cavity brushing, minimal specimens, as few as four viable cells.^3^, ^4^ Brewington et al^14^ generated long‐term cultures from nasal brushing samples. Two groups reported the use of CR technology to expand cells from liquid biopsies (blood and urine samples).^15^, ^16^ In many cases, CR works well for brushing samples, needle biopsies, and other minimally invasive samples from endoscopic exams, especially samples from respiratory tract, digestive tract, and genital‐urology tract. Figure 1 shows broad tissue types and function platforms.

### 2.3 CR is general

CR method is generally applicable to many tissue types including nasal, oropharynx, pharynx, laryngeal, trachea, bronchial, lung, breast, skin, kidney, prostate, bladder, salivary gland, oral cavity, esopharyngx, stomach, small intestine, colon, liver, and neuroendocrine or endocrine tissues^3^, ^4^, ^17^, ^18^, ^19^, ^20^, ^21^, ^22^, ^23^, ^24^, ^25^, ^26^, ^27^, ^28^, ^29^, ^30^, ^31^, ^32^, ^33^, ^34^, ^35^, ^36^, ^37^, ^38^, ^39^, ^40^, ^41^, ^42^, ^43^, ^44^, ^45^, ^46^ (Figure 1). CR is also applicable to several mammalian species such as horse, dog, mouse, rat, ferret, and cow.^47^, ^48^, ^49^, ^50^, ^51^, ^52^ Besides the generation of primary cancer/normal cell lines, CR can be used to establish xenografts^3^, ^4^ and PDX cell lines^53^ and it can also generate cell cultures from PDX and organoids.^38^, ^54^, ^55^

### 2.4 CRCs can be generated from cryopreserved biopsies

When we started CR culture, we also determined that CR technology allows the propagation of cells from cryopreserved tissue specimens.^56^ CR cells could be frozen at −80°C or liquid nitrogen for long‐term storage and then thawed out when needed.

### 2.5 CRCs can be genetically manipulated with CRSPR editing or lentiviral infections

CRCs can be genetically manipulated with gene‐editing technology,^57^, ^58^ which suggests the potential usage in the studies of molecular mechanism and gene therapy. Jonsdottir et al^59^ established CRCs and ALI cultures from both upper and lower airway to study the host innate immune response to human coronavirus 229E (HCoV‐229E) and human respiratory syncytial virus (RSV) after gene manipulations. This study demonstrated that the expression of host gene can be regulated via inducible shRNA knockdown, and these genetic modifications do not influence the host innate immune response, thereby unlocking a unique potential for molecular characterization of virus‐host interactions in human airway epithelium.

### 2.6 CR technology is simple and cheap

Originally, the CR used irradiated mouse fibroblast cells (swiss mouse 3T3, J2 clone) and the Rho‐associated kinase inhibitor (Y‐27632) to propagate epithelial cells.^3^, ^5^, ^6^ A few improvements have been used to simplify protocols using J2 conditioned medium,^3^, ^5^, ^6^ hypoxia condition (1‐2% O_2_),^60^ and combination with mTOR or TGF‐beta, and SMAD inhibition^61^, ^62^, ^63^ in the presence of Y‐27632. CR technology is simple and cheap as there is no need for expensive reagents as matrigel for organoids, and robust as 1 × 10^6^ cells can be generated from a needle biopsy within 7 days, and rapid as the whole populations of cells can be reprogrammed within 2 days instead of needing clonal selection. Figure 1 shows a diagram of normal cell cultures in CR conditions for long‐term cultures and 3D (ALI, air‐liquid interface; LLI, liquid‐liquid interface; and organoids) conditions for ex vivo models of airway epithelial cells.

## 3 CRCs MAINTAIN THEIR LINEAGE FUNCTIONS

**Figure 2:**
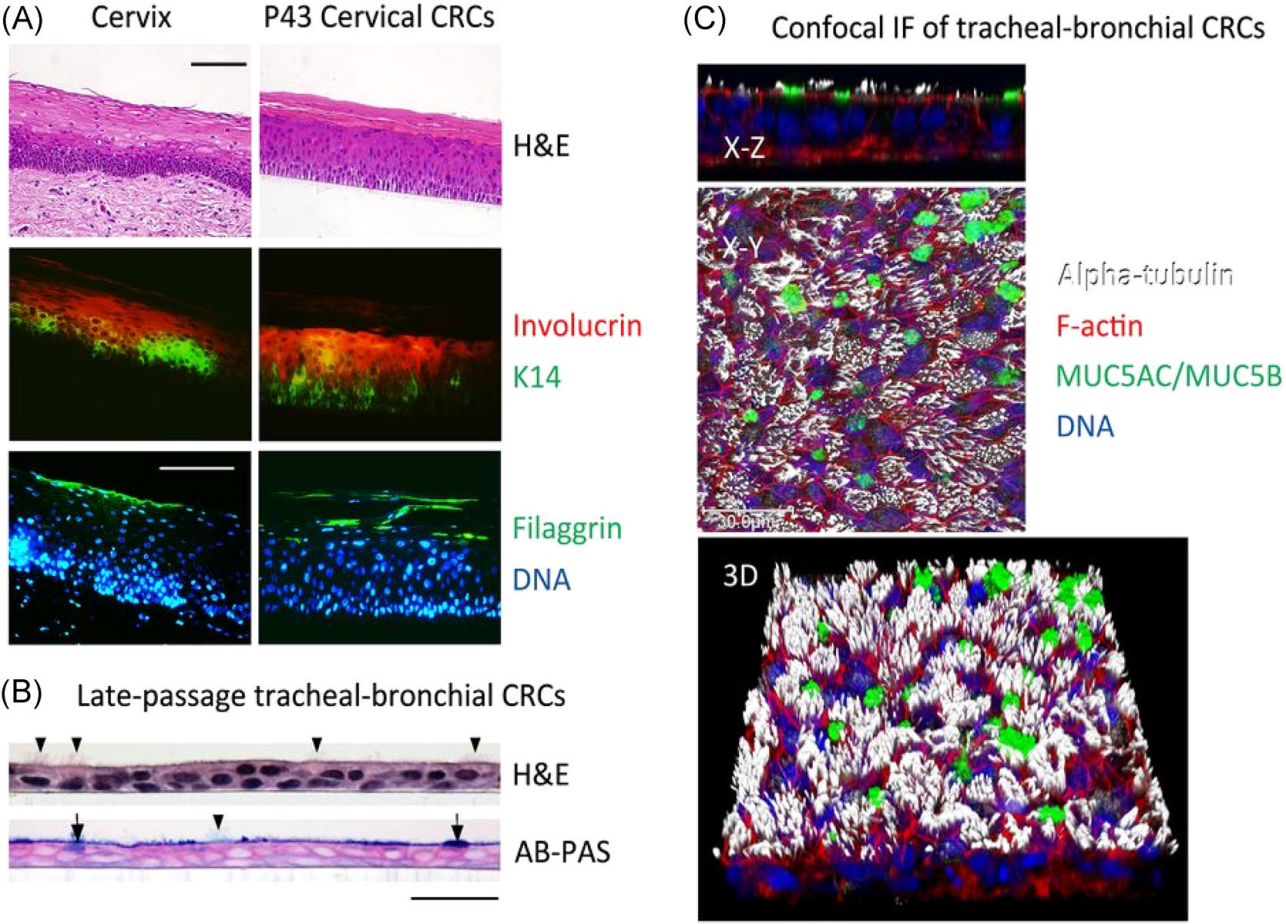
Tissue‐specific differentiation of normal CRCs under ALI cultures. A, H&E histology staining of normal cervix, ALI culture of normal cervical CRCs. Serial sections were stained with primary antibodies against K14, involucrin and filaggrin, and with fluorescent secondary antibodies and Hoechst dye 33258 for DNA. B, Histological sections of ALI cultures of airway CRCs. Sections were stained with H&E or a combination of alcian blue and periodic acid‐Schiff reaction (AB‐PAS). Note the presence of ciliated cells (arrowheads) and mucus‐producing cells (arrows). C, Confocal microscopy of airway CRCs that were differentiated in ALI culture, fixed and fluorescently labeled with phalloidin (F‐actin), Hoechst dye 33342 (DNA), or antibodies demonstrating the presence of cilia (alpha‐tubulin) and mucins 5AC and 5B (MUC5AC/MUC5B). An *X*‐*Z* cros section, extended focus *X‐Y* view, and corresponding three‐dimensional (3D) view are shown. ALI, air‐liquid interface; CRC, conditionally reprogrammed cell; H&E, hematoxylin and eosin (adapted from *PNAS* 2012;109(49):20035‐20040 (https://www.pnas.org/page/authors/licenses), 4 December (https://doi.org/10.1073/pnas.1213241109)^4^

As shown in Figure 1, CR technology can be used for the rapid generation and long‐term cultures of multi‐lineage cells from a variety of normal tissues. These normal CRC cultures retain their normal karyotype and differentiation properties. Importantly, CRC cultures are reversible, CR cells can differentiate normally under in vitro 3D (eg, air‐liquid interface [ALI]: open apical culture; organoids: closed 3D cultures) or in vivo (in animal) conditions (Figure 1, right panel). For example, when CR cells from cervical epithelium or tracheal epithelium are placed in an ALI system, the cervical cells form a well‐differentiated stratified squamous epithelium (Figure 2A), whereas the tracheal cells form a ciliated airway epithelium (Figure 2B). These indicate the potential of CRC cells in tissue regeneration, and as a physiological model for virus infections and evaluation of drug toxicity. ALI cultures (Figure 2) of CR cervical and airway cells are uniquely suited as ex vivo models for studies of virus infections, as ALI cultures can be used to faithfully recapitulate in vivo key characteristics of the normal airway or cervical tissues. For CR airway epithelial cells, ALI 3D cultures maintain a normal composition of cell types, polarized cellular and junctional properties of airway tissues, dynamic physiologic processes such as mucus secretion and coordinated ciliary beating, and physiological expression and subcellular localization of characteristic proteins bearing species‐specific sequences.

## 4 CRCs MODELING VIRAL DISEASES

Either CRC cells alone or in combination with in vitro or in vivo 3D cultures have been used in studies of modeling human diseases, for example, lung cancer, COPD, cystic fibrosis (CF), asthma, viral infections, etc, and also for drug screening and toxicity testing, wound‐healing or tissue repair, and gene therapies.^1^, ^2^, ^14^, ^22^, ^24^, ^29^, ^31^, ^32^, ^33^, ^34^, ^35^, ^38^, ^39^, ^41^, ^42^, ^47^, ^50^, ^54^, ^60^, ^64^, ^65^, ^66^, ^67^, ^68^, ^69^, ^70^, ^71^, ^72^, ^73^, ^74^, ^75^, ^76^, ^77^, ^78^, ^79^, ^80^, ^81^, ^82^, ^83^, ^84^, ^85^, ^86^, ^87^, ^88^, ^89^ We recently summarized the applications of CRC/ALI in emerging virus infections of the respiratory tract (VS in press). Organoids, a close 3D culture system, may recapitulate several characteristics of their original tissue types. Inoculation of infectious materials such as viruses can be injected to “holo” structures. While ALI system is relatively easier for the study of viral infections because of an open apical culture system where the release of cytokines, enzymes, expression of cellular or viral genes, viral products or particles can be measured or detected from upper, lower compartments or “tissues.”

### 4.1 Epstein‐Barr virus and nasopharyngeal carcinoma

Epstein‐Barr virus (EBV), first discovered from a Burkitt lymphoma, is a potentially oncogenic herpesvirus, which involves B cell lymphoma and nasopharyngeal carcinoma (NCP). EBV latency and replication have been intensively studied in B cells, as they are easily infected and maintained in vitro. EBV infection becomes a general method to immortalized B cells for a variety of applications. However, the interaction of EBV with epithelial type has been significantly hampered by difficulties in establishing reproducible and robust infection in vitro. Expression of EBV genes including EBER1/2, EBNA1, LMP1, and LMP2 is shown in primary NPC tissues, and these genes may contribute to the initiation and progression of NPC.^90^ One of the major obstacles in the field of EBV and NPC is the scarcity of available NPC lines for research. Most commonly used cancer cell lines in the field are contaminated with HeLa cells.^91^, ^92^ The C666‐1 is the only EBV‐positive cell line extensively used in EBV and NPC research, this cell line was established from an earlier established NPC xenograft,^93^ and is defective for lytic EBV reactivation EBV. However, EBV infection is an essential feature of NPC and it is important to establish NPC cell lines harboring EBV episomes, which are critical tools in understanding EBV in NPC initiation and progression. Researchers from Hong Kong were successfully able to establish two new NPC cell lines (C17 and NPC43) harboring EBV from the C17 NPC xenograft^94^ and a NPC patient^95^ using CR technology. Both new NPC cells exhibit tumorigenicity in mice, and can be induced to undergo EBV lytic reactivation with the production of infectious particles. These new lines represent novel model system for EBV and NPC studies. Temple et al^96^ established 3D “raft” cultures from primary tonsil or gingival epithelial cells and successfully infected them from the apical surface of 3D cultures with either cell‐free virus or EBV‐producing B cells. They observed that replicating EBV spread throughout the suprabasal epithelium with expression of viral latency proteins and the lytic cycle, suggesting an efficient replication of EBV in the stratified epithelium. A few key questions in EBV and NPC field, for example, infectivity of EBV in epithelial cell types (tropism) and unique oncogenic activity in nasopharyngeal epithelial cells, have not been able to be studied because of lack of appropriate normal cell model systems. Thus, these above questions will be studied or may even be answered in the field using combination with CR technology and ALI cultures, in addition to generation of patient‐derived EBV‐positive NPC cell lines using CR technology.^94^, ^95^

### 4.2 Human papillomaviruse and human disease

Human papillomaviruses (HPVs) are associated with several benign and malignant human diseases, including skin or genital warts, and human cancers of vagina, cervix, anus, penis, vulva, skin, and oropharynx. Cervical cancer cell lines such as HeLa (HPV 18 positive), SiHa (HPV 16 positive), Caski (HPV 16 positive), and C33A (HPV negative), have been exclusively used for cervical cancer research and anti‐cervical cancer, or anti‐HPV discovery, while currently there is still no specific therapies available for HPV‐associated lesions including cervical cancer. We identified an effective and unexpected therapy for a patient with aggressive recurrent respiratory papillomatosis (RRP) using patient‐derived CR cells and phenotypical screening.^97^ We also discovered a mutant HPV 11 genome from lesion tissues. Currently, there is no available treatment for HPV lesions because of the unavailability of episomal HPV‐positive cell system. Recently CR HPV‐6‐positive laryngeal cells were used for high‐throughput drug screening at the National Center for Advanced Technology (NCATS).^85^ CR HPV‐6 cells were used for high‐throughput screening against two libraries: (1) the NPC library of >2800 approved drugs; and (2) the MIPE library of >1900 investigational drugs to identify new indications for FDA‐approved drugs or novel candidate drugs at research and development stage, respectively. They identified a total of 13 drugs with significant killing effects in CR RRP cells from two libraries and validated their effects of the drugs using in vitro 2D and 3D models. The 3 (panobinostat, dinaciclib, and forskolin) of 13 drugs have the potential for future therapies of RRP patients. The McBride laboratory discovered that Sp100 may act as a repressor of incoming HPV DNA,^98^ and that original core replication origin (three E2 binding sites located) and additional sequences from the transcriptional enhancer portion of the upstream regulatory region (URR) are required in *cis* for long‐term HPV 18 genome replication using CR keratinocytes.^99^ Therefore, CR can be used for the generation of HPV‐positive benign or malignant human lesions, anti‐HPV discovery, and biology of both high‐ and low‐risk HPVs through native viral infections or HPV DNA transfections in CR host epithelial cells.

### 4.3 Adenovirus

Adenovirus infection is common in the patients with pre‐existing respiratory diseases, for example, Asthma, COPD or CF, etc. suggesting that inflammatory factors may modulate virus infection to airway epithelial cells. Coxsackievirus and adenovirus receptor (CAR), a protein, encoded by the CXADR gene, is a receptor for coxsackie viruses (group B) and adenoviruses (subgroup C). CAR is the primary receptor for efficient virus attachment. Kotha et al^73^ first established polarized ALI cultures using CR airway cells and Calu‐3 cells and discovered that IL‐8, a pro‐inflammatory cytokine, increased the levels of β_1_ integrin (a viral coreceptor) at the apical surface. Coreceptors integrins may facilitate adenovirus endocytosis and endosomal escape. They also showed that physiological level (at 100 ng/mL concentration) of IL‐8 stimulate the expression and localization of the primary apical adenovirus receptor, CAR^Ex8^, in polarized human airway epithelia, resulting in the enhanced Ad5 FK‐sensitive AdV infection from the apical surface of ALI cultures. However, IL‐8 did not affect the expression of total CAR because CAR^Ex7^ is a predominant form, indicating that these two isoforms of CAR may have different functions within a biological epithelium. They also found that IL‐8 acutely stimulates maximal expression of CAR^Ex8^ between 4 and 12 hours. These suggest that CAR^Ex8^ may be essential for facilitating early innate immune responses of the host to virus infection. Thus, CR technology allows generation of airway cells from healthy donors or patients with pre‐existing conditions to study the adenovirus infection in host airway cells. CR/ALI combination will provide a unique physiological or pathological system for host‐virus interactions.

### 4.4 Human rhinovirus infection and asthma

Airway cells or tissue specimens obtained from a bronchoscopy procedure from patient's lower airway are important for disease diagnostics, and basic or clinical research. However, this procedure may cause bronchospasms, bleeding, and infection. There is an urgent need for a noninvasive or minimally invasive approach such as nasal brushing to study these airway diseases. Thus, a key question is whether nasal epithelial cells are ideal surrogates for tracheal or bronchial epithelial cells. Roberts et al^100^ established parallel nasal and bronchial epithelial cells using CR technology. These cells were treated with IL‐13 for 2 weeks on the first day of ALI cultures, or for 3 days on day 11 of ALI cultures. These represented chronic and acute system, respectively. On the viral infection day, the cells were washed with DPBS and then infected with human rhinovirus 16 (HRV16). They first demonstrated the mucociliary differentiation of bronchial and nasal epithelial cells at the ALI cultures. Then, they focused on several highly relevant indicators to acute exacerbations to HRV infection and asthma: interferon γ‐induced protein 10 (IP‐10), eotaxin 3, a chemokine for eosinophilic inflammation, viral load and an antiviral gene Mx1, one of the interferon‐stimulated genes. Their findings support that nasal and bronchial epithelial cells have similar responses to HRV16 infection and IL‐13 treatment. Wesolowska‐Andersen et al^101^ generated CR human tracheal airway epithelial cells from three donors. After 21 days of growth at ALI cultures, paired differentiated cells from each donor were infected with mock control or with HRV‐A16 (human rhinovirus A16). Then, they demonstrated similar changes of the transcriptome for in vitro viral infection as those in vivo. For in vivo analysis, they screened 92 asthmatic and 69 healthy children without viral illness using qPCR for common respiratory viruses and for two known genes (CCL8/CXCL11), upregulated in viral infections. They found 21 viral qPCR‐positive and 2 suspected virus‐infected individuals with expression of CCL8/CXCL11. Their dual RNA‐seq results demonstrated that viral infection without illness may determine the airway function of these individuals by cellular remodeling, driving airway infiltration of immune cell, and alterations of asthmogenic expression. As shown in Figure 1, CR technology allows to generate long‐term stable cultures from anatomic sites of respiratory tract for viral infections in 2D or further establishment of polarized ALI cultures as a physiological condition for virus‐host interactions.

### 4.5 Human parvovirus

Human bocavirus 1 (HBoV1) often infects children and causes acute respiratory tract illness, such as pneumonia, induces, asthma exacerbations, and/or bronchiolitis, and some are life‐threatening.^102^ It has been shown that HBoV1 may infect well‐differentiated or polarized human primary airway epithelium cultured at ALI cultures.^103^ To overcome difficulties such as variability and the low yield of human primary cells, Qiu's group obtained a large amount of airway epithelial cells that used feeder‐based or feeder‐free CR technologies.^3^, ^6^, ^61^, ^104^, ^105^ They first established CR cells and ALI cultures (non‐dividing airway epithelial cells) and inoculated ALI with human parvovirus HBoV1. They demonstrated that HBoV1 infection of ALI cultures induces a DNA damage response (DDR), thereby facilitating viral genome amplification. They also discovered that Y‐family DNA repair polymerases, Pol η and Pol κ, are involved in HBoV1 genome amplification in ALI system. This is the first report to show that parvovirus DNA replicates in non‐dividing cells autonomously.^104^ Then, they also discovered that HBoV1 infection activates antiapoptotic proteins, thereby suppressing apoptosis but promoting pyroptosis.^105^ Thus, CR‐coupled ALI system may serve as a physiological model for study interactions of HBoV1 and host cells, and a system for antiviral discovery as well.

### 4.6 Herpes simple virus

Zhu et al^106^ studied herpes simple virus‐2 (HSV‐2) infection in CR and ALI‐cultured normal vaginal epithelial cells. Herpes simplex virus type 2 (HSV‐2) may infect human genital mucosa and establish latent status lifelong. They first established long‐term human normal vaginal epithelial cells (HNVEC) using coculture‐based CR technology. These cells that exhibited a normal diploid karyotype and formed well‐defined and polarized spheres in 3D Matrigel cultures, a normal response to DNA damage stimulus, did not form colonies in soft agar assays. Then, they reconstructed 3D vaginal epithelium using ALI culture containing the basal and apical layers with expression of epithelial markers as the original vaginal tissue. Finally, they infected HSV‐2 (G strain) at apical layer of ALI vaginal epithelium, and observed typical pathological effects spreading from the apical layer to basal layer with expression of a viral protein. ALI cultures of vaginal cells can also be used in a broad range of reproductive immunology research, such as how vaginal cells respond to viruses or other microbes in controlled and physiological conditions (hormonal microenvironments).^107^

### 4.7 Zika virus

Fink et al^108^ also established CR cells from routine vaginal repair surgeries or hysterectomies and studied how the antiviral drug (Arbidol) inhibits the Zika virus. They first generated CR culture of primary, untransformed epithelial cells from ex vivo tissues, discarded following routine vaginal repair surgeries or hysterectomies. They also made ZIKV pseudovirus (PsV) from HEK‐293T cells cotransfected with a DNA‐launched West Nile virus (WNV) replicon expressing EGFP and a plasmid expressing ZIKV C‐PrM‐E (envelope). To focus on viral entry mediated by the ZIKV envelope glycoprotein, they infected Vero cells with the ZIKV PsV. PsV entry could be blocked by bafilomycin A1 (a known inhibitor for endosomal acidification). Their results demonstrated that ARB pretreatment was able to prevent PsV infection to Vero cells. However, ARB was less effective in inhibition of PsV infection, when ARB was added to cultures after removal of the virus inoculum, suggesting that ARB blocks an early step of life cycle of the Zika virus, and may also have postentry effects. Then, they showed that CR cells (vaginal, endocervical, and ectocervical cells) were robustly infected by the ZIKV virus. ARB treatment resulted in significant inhibition of synthesis of viral protein and RNA. Importantly, the dose of ARB (20 μM) was not toxic to all these cell types.

### 4.8 Distal airway epithelial cells and influenza virus

Imai‐Matsushima et al^109^ generated a long‐term culture of distal airway epithelial cells (DAECs) from both human and chicken. As expected and reported from other studies, the removal of feeder layers induced a strong inflammatory response and differentiation into mixed airway cell types. They also found that small molecules and growth factors at the end of the expansion phase may induce differentiation of DAECs into alveolar type II (ATII) cells, then eventually transdifferentiation into type ATI cells. They infected human and chicken DAECs with different IAV strains: A/WSN/1933 (H1N1), A/England/195/2009 (H1N1pdm), A/Panama/2007/1999 (H3N2), A/Vietnam/1203/2004 (H5N1), and A/Mallard/Germany/439/2004 (H3N2). The results showed that A/WSN/1933(H1N1) (the lab‐adapted), A/Panama/2007/1999(H3N2) (the seasonal), and A/England/195/2009(H1N1pdm) (the pandemic), generated as a triple reassortment of human, porcine, and avian viruses, replicated very well, even better in chicken DAECs. However, A/Mallard/Germany/439/2004(H3N2) exhibited relatively low replication efficiency (especially in human DAECs), A/Vietnam/1203/2004 (H5N1) (the highly pathogenic) replicated equally well in both species. These model systems allow physiologically relevant research on various human and zoonotic lung diseases, also support siRNA transfection, enabling the application of advanced molecular techniques, for example, gene editing, on primary DAECs.

### 4.9 Hepatocyte cultures and liver diseases

Primary liver cancer is the 6th most frequent cancer type globally with high mortality, partially due to the lack of effective therapeutic options. The leading cause of liver cancer is cirrhosis due to viral hepatitis (HBV and HCV), aflatoxin, nonalcoholic fatty liver disease, or alcohol. The most common type is hepatocellular carcinoma (HCC). HCC development is often accompanied with unique and continuous genetic and epigenetic alterations. Therefore, the absence of a personalized and reproducible human model reduces the ability to determine the potential of candidate treatments. Wang et al^45^ generated CRCs from primary HCC tumor specimens with a success rate of ~55%. They confirmed the expression of the tumor‐specific marker α‐fetoprotein and the proliferative ability of cells following cycles of cryopreservation and resuscitation. These CR HCC cells will be extremely important for HCC, HBV, and HCV. Su et al^67^ reported primary hepatocytes that were grown from 6 of 11 specimens under CR conditions, which are genetically identical with original tissues and retain strong CYP3A4, 1A1, and 2C9 activities. The same group also established CR cell culture from a patient with ornithine transcarbamylase deficiency (OTCD).^42^ OTCD CR cells retained native CYP3A4, 1A1, 2C9 activities and albumin secretion function at early passages. Under 3D culture environment, low urea production and hepatocyte marker were also detected. These reports indicated that CR technology can be used to generate normal and disease CR hepatocytes that may be useful for studies of hepatitis‐associated viruses, such as HBV, HCV, HDV, and CMV.

### 4.10 Gastrointestinal diseases

Currently, there are no reports regarding studies of viral infection in CRCs from gastrointestinal (GI) tract. There are several common viral diseases that cause health problems worldwide. It is important to discuss the potential applications of CR technology in GI viral diseases. For example, severe acute respiratory syndrome coronavirus‐2 (SARS‐CoV‐2) may infect small intestine epithelial cells because of high levels of ACE2 expression. Here, we discuss the applications of CRCs from GI tract in human diseases.

#### 4.10.1 Esophageal cells

Jensen et al^64^ first discussed the generation of CRCs from pediatric esophageal tissues for tissue engineering and esophageal disease studies. These CRCs do not need to be sorted or purified and can return to a mature epithelial state after removal of CR conditions. CR esophageal cell‐based screening assays may help revolutionize the treatment of pediatric esophageal diseases like eosinophilic esophagitis. For the patients with a congenital defect, disease, or esophageal injury, these CR esophageal cells can potentially be used for implantation to repair or replace the affected region. Sayel et al^110^ established patient‐derived esophageal epithelial cell lines using the CR method. They found that esophageal CRCs maintained their phenotype during passages. They also found differences of profiling integrin and gene expression in EoE‐active compared with normal controls and EoE‐Remission patients. Once these cells were stimulated with antigens, esophageal CRCs expressed MHC class II on their surface, and when cocultured with autologous T‐cells, there was increase in IL‐6 and TNF‐α secretion in EoE‐active patients compared with controls.

#### 4.10.2 Gastric cells

Han et al^111^ designed 3H11‐CARs for modified T‐cell therapy of gastric cancer. They first confirmed that the single‐chain variable fragment (scFV) of the mAb 3H11(scFV‐3H11) exhibited same activity as the natural antibody. Then, they tested that scFV‐3H11 CAR‐T cells were able to kill cancer cells with increased interleukin‐2 and interferon‐γ secretion in vitro. Finally, they verified that scFV‐3H11 CAR‐T cells reduced the tumor burden in mice with cancer cell lines and patient‐derived CR gastric cancer cells.

#### 4.10.3 Intestine cells

Moorefield et al^82^ created 2D mouse intestinal epithelial monolayers from genetic mouse models for functional analysis using CR technology. These CR intestinal cells were used for functional analysis under 3D matrigel organoid culture and on transwell inserts. The results turned out that CRCs from the cystic fibrosis (CF) mouse model CFTR ∆F508 failed to respond to forskolin, a CFTR activator, in 3D matrigel and transwell cultures. CRCs from the ApcMin/+ mouse intestinal cancer model grew faster than those from wild‐type mouse under both CR condition and matrigel 3D organoid culture. The results suggest that CR intestine cells is a useful model system to obtain large amount of genotype‐specific epithelial cells for studies of molecular mechanisms of diseases and identification novel therapeutics.

#### 4.10.4 Colorectal cell

Wang et al^112^ generated CRCs from colorectal cancer patients and evaluated drug response using high‐throughput screening of CRCs in vitro and CRC‐derived xenografts (CDX) *in vivo*. They discovered synergistic inhibitory effect of the EGFR and MEK or CDK4/6 inhibitors to treat colorectal cancer, suggesting that the novel combination of CR cultures and their corresponding CDX models had great potential in individualized therapy and drug discovery.

Above reports suggest that CR technology can be used to propagate normal and diseased cells from GI tract that are potentially useful for studies of GI viral infections and fecal‐mouth transmission. As epithelial cells in the small intestine express high levels of ACE2, the CR intestine‐epithelial cells will be helpful for SARS‐CoV‐2 study.

### 4.11 Genital‐urological diseases

As described above, there are no reports regarding studies of viral infection in CRCs from genital‐urological (GU) tract. We also discuss the potential applications of CR technology in GU viral diseases. For example, SARS‐CoV‐2 may infect and damage kidney because of high levels of ACE2 expression.

#### 4.11.1 Kidney

Saeed and colleagues^113^ established multiple CRCs from different regions of each tumor from four patients with RCC (renal cell carcinoma) and verified their clonal relationship and the parental tumors. They then performed a comprehensive drug‐sensitivity testing for all CR clones. The results indicated that the CR RCC cells retained cancer‐specific copy number alterations and somatic mutations as those in the corresponding original tumors. Drug‐testing demonstrated sensitivity in the CR RCC cells to conventional RCC drugs, such as the mTOR‐inhibitor temsirolimus and novel potential agents.^113^ Finally, they also studied the response profiles of CR RCC cells from different regions (primary tumor, invasive vena cava, and adrenal metastasis) in a patient's tumor tissues.

#### 4.11.2 Bladder

Kettunen et al^114^ generated CRCs from bladder cancer (BC), compared profiles of genetic and protein expression in CRCs to those in primary tumors, and performed personalized drug‐sensitivity screening. The results demonstrated that these CRCs were sensitive to conventional agents (eg, taxanes, proteasome, and inhibitors of topoisomerase) and standard BC chemotherapy drugs (eg, cisplatin and gemcitabine).^114^ Jiang et al^16^ successfully established CRCs from BC patients’ urine samples. The overall success rate of urine CRCs was 83.3%. Then, they validated similar response of the urine CRCs and patients’ responses.

#### 4.11.3 Prostate

Timofeeva et al established matched normal and tumor CRC cultures from a patient's prostatectomy specimen. Only tumor‐derived CRCs formed tumors in SCID mice, demonstrating maintenance of the critical tumor phenotype. They also demonstrated that both normal and tumor cells predominantly expressed high levels of basal cell markers and low levels of luminal markers under CR conditions. When injected into SCID mice, the expression of luminal markers increased significantly, while basal cell markers dramatically decreased. Tricoli et al reported a novel filter‐based multidimensional culture platform, that is, transwell‐dish culture method,^115^ which enabled stratification of normal and tumor CRCs. Choudhary et al^55^ established an engineered bone tissue model integrated by 3D‐networked primary human osteocytes with CR prostate cancer cells. They found that SOST (Sclerostin) was widely expressed in osteocytes within the 3D tissue cultures, but SOST expression was significantly decreased when osteocytes were cocultured with CR cancer cells.

These suggest that CR technology can be used for expansion of normal and tumor cells in GU system. These cells may serve an *ex vivo* models for studies of viral infections in genital‐urology, for example, SASR‐CoV‐2 induced injury of the kidney.

## 5 SARS‐CoV‐2 AND CORONAVIRUS DISEASE‐2019

**Figure 3:**
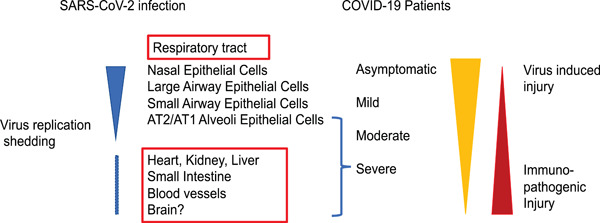
Proposed diagram of SARS‐CoV‐2 replication and immunopathogenic injuries for COVID‐19 patients. COVID‐19, coronavirus disease‐2019; SARS‐CoV‐2, severe acute respiratory syndrome coronavirus‐2

World Health Organization (WHO)^116^ declared the coronavirus disease‐2019 (COVID‐19) or SARS‐CoV‐2 infection outbreak as a “public health emergency of international concern,” and characterized COVID‐19 as a “pandemic” on 30 January and 11 March 2020, respectively.^117^ Patients with COVID‐19 have ranged from asymptomatic (or very mild), mild, moderate, severe, to critical severe illness resulting in death.^118^, ^119^, ^120^, ^121^, ^122^, ^123^, ^124^, ^125^, ^126^, ^127^, ^128^, ^129^ While around 80% of COVID‐19 patients have mild symptoms, some patients may progress to pneumonia even to multiorgan failure involving the lung, heart, and kidney.^118^, ^119^, ^120^, ^121^, ^122^, ^123^, ^124^, ^125^, ^126^, ^127^, ^128^, ^129^ The rate of death among confirmed patients is estimated to be 5% but varies by age and other health conditions.^118^, ^119^, ^120^, ^121^, ^122^, ^123^, ^124^, ^125^, ^126^, ^127^, ^128^, ^129^ The mechanisms of how SARS‐CoV‐2 infects human airway epithelial cells and also causes severe multiorgan failure are largely unknown. COVID‐19 patients at the early stage of or asymptomatic or mild symptomatic patients usually have rapid replication of viruses at upper airway. Whether or not SARS‐CoV‐2 spreads to lower airway track is due to virus‐host interaction, innate response, and local immune response. Severe COVID‐19 patients with multiorgan injury are usually due less to virus replication‐based direct injury and more to immunopathogenic injuries (Figure 3).

SARS‐CoV‐2 and SARS‐CoV share the same functional host‐cell receptor ACE2,^130^, ^131^, ^132^, ^133^, ^134^ and that SARS‐CoV‐2 possesses crucial amino acid residues for ACE2 binding.^133^ ACE2 predominantly expresses in vascular endothelial cells, kidney and heart tissues, small intestine, and testes.^135^ Recently, two reports demonstrated large amounts of SARS‐CoV‐2 in the upper airway and saliva.^116^, ^123^ Human‐related physiological models are urgently needed for these studies of body site‐specific or tissue‐specific viral replication, innate immune response, and infectivity. As a functional and biological system, CR coupled with ALI/LLI culture will facilitate these studies and development of novel therapeutics (Figure 1). Indeed, early study on SARS‐CoV indicates that host cell differentiation or polarized epithelium and expression of ACE2 are both important for the susceptibility of human airway epithelia to SARS‐CoV viral infection.^136^ Indeed, Baric lab at UNC has used for ALI cultures of human airway epithelial cells (HAEs) for functional drug screening of SARS‐CoV and SARS‐CoV‐2.^137^, ^138^ To overcome the difficulties with stable source and expense of primary human normal cells and variability of donors, CRCs from airway, GI, GU tracts in combination with ALI or LLI will be a better choice for physiological systems for SARS‐CoV‐2 studies (Figure 1).

## 6 SUMMARY

Owing to the limitations of current cell line and animal models, there is an urgent need for human physiological cell models for the study of viral infections and discovery of antiviral drugs. Here, we summarized the long‐term cultures for human normal epithelial cells from respiratory tract, GI, and GU tracts using CR technology. Their cultures provide a stable source for normal cells from individuals and populations (race, age, gender, geography, etc.); CRC‐coupled ALI/LLI/Organoids technologies (Figure 1) may serve as *ex vivo* physiological models for host‐virus interactions and human viral disease, especially emerging and re‐emerging virus infections. These will facilitate studies of virus entry, innate immune responses, viral replications, and drug discovery.

## DISCLOSURE

Several patents for conditional reprogramming technology has been awarded to Georgetown University by the United States Patent Office. The license for this technology has been given to Propagenix for commercialization. The inventor, XL, and Georgetown University receive potential royalties and payments from Propagenix.
